# Relationship Between Unintended Pregnancy and Antenatal Care Use During Pregnancy in Hadiya Zone, Southern Ethiopia

**Published:** 2019

**Authors:** Desta Erkalo Abame, Muluembet Abera, Amanuel Tesfay, Yonas Yohannes, Dejene Ermias, Terefe Markos, Gelila Goba

**Affiliations:** 1- Haramaya University, College of Health and Medical Sciences, School of Public Health, Harar, Ethiopia; 2- Jimma University, Institute of Health, Faculty of Public Health, Jimma, Ethiopia; 3- East Badawacho, District Health Office, Hadiya Zone, Ethiopia; 4- Wolaeta Sodo University, College of Health Sciences, Wolaeta, Ethiopia; 5- Department of Obstetrics and Gynecology, University of Illinois at Chicago, Chicago, Illinois, USA

**Keywords:** Antenatal care, Ethiopia, Hadiya, Unintended pregnancy

## Abstract

**Background:**

Unintended pregnancy has direct relation with poor utilization of maternal health care services and also associated with unhealthy behaviors during pregnancy. Few studies have examined the association between unintended pregnancy and maternal health behaviors during pregnancy in developing countries including Ethiopia. The purpose of the study was to determine the association of unintended pregnancy with use of antenatal care during pregnancy among pregnant women in Hadiya zone, southern Ethiopia.

**Methods:**

Community based cross sectional study design was employed in Hadiya zone, southern Ethiopia in 2017. 748 pregnant mothers were included using single population proportion. Study participants were selected by simple random sampling technique. A structured interviewer administered questionnaire was used to collect data. Descriptive, bivariate and multivariate logistic regression was employed to identify the independent effect of unintended pregnancy on the outcomes of interest. The level of significance was confirmed if p-value was less than 0.05.

**Results:**

More than one third (36.2%) of women reported unintended pregnancy. Unintended pregnancy was significantly associated with use of antenatal care. Women with unintended pregnancy were 69% less likely to receive ANC (AOR=0.31, 95% CI; 0.21–0.46) and were four times more likely to have late ANC initiation (AOR=4.40, 95% CI; 1.70–11.40) during pregnancy as compared to counterparts.

**Conclusion:**

This study finding showed an association between unintended pregnancy and ANC use during pregnancy. Women with unintended pregnancy were less likely to use antenatal care and more likely to delay initiation of antenatal care. Longitudinal studies are recommended on relationship between unintended pregnancy and ANC use.

## Introduction

Pregnancy is a happy event for the woman, husband, family, and community when it is wanted or intended. But millions of women around the world become pregnant unintended. Unintended pregnancy is when it is either mistimed (That is, they occurred earlier than desired) or unwanted (That is, they occurred when no children, or no more children were desired) at the time of conception (1).

Women across the world experience unintended pregnancies irrespective of their development status. Globally, in 2014, approximately 213 million pregnancies occurred annually, out of which, 40% were unintended. Unintended pregnancy is 36% higher in developing countries than developed countries. In Africa, from a total of 53.8 million pregnancies, 35% were unintended pregnancy in the same year (2). Of these, 50 percent ended in abortion, 13 percent ended in miscarriage, and 38 percent resulted in an unplanned birth (2). The situation is not different from less developed countries in Ethiopia; women suffer from problem of unintended pregnancy. According to Guttmacher Institute’s report in Ethiopia, in 2014, about 4.93 million pregnancies occurred annually, out of which 1.9 million (38%) were unintended pregnancy (3).

About 830 women die from preventable causes related to pregnancy and delivery around the world every day. Almost all maternal deaths (99%) occur in developing countries (4). In Ethiopia, the maternal mortality ratio was 412 per 100,000 live births according to Ethiopian Demographic Health Survey (EDHS 2016) (5). One target under Sustainable Development Goal (SDG) 3 is to reduce the global maternal mortality ratio to less than 70 per 100 000 births (6). Therefore, reducing maternal mortality is closely related to prevention of unintended pregnancy (1).

Unintended pregnancy results in unsafe abortion that is one of the direct causes of maternal mortality and morbidity (7). It leads to induced abortions that can have deleterious consequences for women living in countries where abortions are generally unsafe (8). Preventing unintended pregnancies through effective family planning could avert about 30% of maternal deaths (9).

Pregnancy is a crucial time to promote healthy behaviors and parenting skills. World Health Organization (WHO) recommends that adequate antenatal care for a normal pregnancy that has no complications should comprise four Antenatal care (ANC) visits, with the first occurring within the first trimester (10). Antenatal care provides an opportunity to deliver interventions for providing health education, improving maternal nutrition and encouraging skilled attendance at birth. Early enrollment in ANC is a widely accepted and recommended behavior for pregnant women to improve pregnancy outcome and late enrollment is viewed as a behavior that places women at increased risk of poor pregnancy outcome.

Pregnancies that are unintended result in unhealthy behaviors or continue unhealthy behaviors during pregnancy (11). Thus, unintended pregnancy has direct relation with poor utilization of maternal health care services during pregnancy like delayed initiation of, or low attendance at antenatal care visits (12–15). Even though the studies were conducted in developed countries, limited findings from developing country studies suggested that unintended pregnancy has association with unhealthy maternal behaviors during pregnancy such as use of illicit drugs, smoking, and drinking alcohol (11).

Besides, women with unintended pregnancies have less attention to pregnancy related complications. And they have low social support and lower scores for self-care behaviors such as use of supplements (Folic acid or multivitamin), vaccination and nutrition (16). Consequently, these problems increase obstetric complications such as unfavorable pregnancy outcome, maternal morbidity and mortality, premature birth, low birth weight, neonatal death, and infant abuse (11).

There are few literatures that focus on the association of unintended pregnancy and antenatal care utilization in developing countries, particularly in Ethiopia. Thus, this study aimed to examine whether unintended pregnancy influences antenatal care utilization during pregnancy.

## Methods

### Study setting and design:

A community based cross sectional study design was conducted in Hadiya zone from March 13, 2017 to April 13, 2017. Hadiya zone was divided into 10 rural Woreda and two administrative towns with total of 329 kebeles from which 303 of them rural and 26 of them were urban. Hadiya zone hosts a total of 1,573,841 populations with a total area of 3542.66 *Km*^2^. In the zone, the contraceptive prevalence rate was 49% and ANC coverage was about 83% (18). The study was conducted from March 13 to April 13, 2017.

The sample size for prevalence of unintended pregnancy was determined using single population proportion formula. The following assumptions were used at a 95% confidence level and 5% margin of error, and p is the proportion from a study in southwestern Ethiopia (p=35%) (12). Considering the 10% non-response rate and design effect of 2, the final sample size was 770.

Multi-stage stratified sampling technique was used. Hadiya zone was stratified as rural districts and town administrations. One town administration and 3 districts were randomly selected from among 2 town administration and 10 districts, respectively. Three districts namely Gibe, Misha and Mirab Badewacho were randomly selected by lottery method for cost and logistic reasons. In the same way, thirteen kebeles (5 from Gibe, 5 from Misha and 3 from Mirab Badewacho) were selected. Three kebeles from Hossana town administrations were included in the study to represent the urban communities. A total of sixteen kebeles from both rural and urban districts were selected by lottery method. The list of pregnant mothers was obtained by conducting census in each selected kebele. Based on population size, a sampling frame which enlists all eligible pregnant mothers was prepared and 770 women were randomly selected to be included in the study and interviewed in their home by using health extension workers. Kebele is the smallest administrative unit in Ethiopia.

The data were collected using a pre-tested structured interviewer administered questionnaire which was developed from EDHS 2016 and other similar literatures. The questionnaire had sociodemographic and socio-economic access to reproductive health services, women’s decision making and maternal health behaviors like ANC use and factors influencing utilization of ANC service. It was translated from English to local language (Hadiyisa) and back to English. Face validation of questionnaire was determined. Ten data collectors and three supervisors, who were qualified with Diploma in nursing and BSc. in public health, were recruited. The data collectors and the supervisors were trained for two days on questionnaire, approach to the interviewees, details of interviewing techniques, respect and maintaining privacy and confidentiality of the respondents. The collected data was checked for completeness, accuracy, clarity and consistency by the principal investigator.

### Measurements:

ANC use refers to use of antenatal care during this pregnancy. Women were asked whether they had used ANC during current pregnancy. The variable was measured by binary variable “yes” for those who use and “no” for not using. Moreover, information was collected on time of initiation of ANC visits to determine whether women started early within the first trimester or initiated late in the second trimester and the third trimester. WHO recommends adequate care for a woman without complications including four ANC visits, with the first visit occurring in the first trimester or before 12 weeks of gestation but not later than 16 weeks (10).

The main explanatory variable was pregnancy intention, which was measured by asking a woman’s desire about her pregnancy at the time she became pregnant. The questions and the answers were as follows; at the time you became pregnant, did you want to become pregnant then, did you want to wait until later, or didn’t you want to have any (more) children at all? The answers were; 1) wanted then intended, 2) wanted to happen later mistimed, 3) did not want at all unwanted. Unwanted and mistimed pregnancies were then grouped together as unintended pregnancies.

Wealth index was used as a measure of socio-economic status of mothers. It was calculated from ownership of the following household resources including radio, television, electricity, bicycle, motorcycle, car, type of floor, type of wall material, type of roof material, toilet facilities, farm land, and of domestic animals such as cattle, sheep, goats, and mule. Five principal components with eigenvalues greater than one were summed to obtain wealth index values after Principal Component Analysis (PCA) was run (18,19). The resulting index was then divided into three categories representing poor, middle and wealthy.

Women’s participation in decision making was measured by composite index composed of eight questions. The women were asked “who in her family usually has the final say on the following decisions” of 1) use family planning, 2) number of children, 3, obtaining health care for yourself, 4) visits to family or relatives and 5) large household purchases (20, 21). Then a composite index of women’s autonomy in household decision making was obtained. Each autonomy indicator was coded as a binary variable (0, 1) where 0 represents a low level of decision making and category 1 represents a relatively high level of decision making (Decisions were made by either woman alone or with husband jointly). Based on these values, the overall score was found to be 8. Therefore, those women who scored half of the total score, *i.e*. 4 and above, were considered as participated in household decision otherwise not participated in household decision and coded 1 and 0, respectively.

### Data processing and analysis:

The data on each coded questionnaire were entered into Epidata version 3.1. Then, the entire data were exported to SPSS version 21 statistical packages for analysis. Descriptive analysis was done to compute frequencies, percentage and cross tabulations. Bivariate analysis was performed to select variables for multivariate analysis. Hence, variables with p-value <0.25 in the bivariate analysis were taken as candidates for multivariate analysis. But, statistical significance was tested at the level of 5% at the multivariate level. Finally, multivariate logistic regression analysis was performed to identify the independent effect of pregnancy intention on the outcomes of interest after controlling other possible confounding variables. Adjusted odds ratios with 95% CI were reported.

### Ethical consideration:

Ethical approval was obtained from ethical review committee of Jimma University, Institute of Health. Support letter was obtained from department of population and family health. The necessary permission was obtained from Hadiya zone health department, and selected Woreda health offices and kebele administrative offices. All the study participants were informed about the purpose of the study, their right to refuse and assured confidentiality and informed verbal consent was obtained prior to the interview.

## Results

### Sociodemographic characteristics:

Out of 770 eligible pregnant women, 748 women were interviewed making a response rate of 97%. The respondents mean age was 27.34 (SD±4.4). A majority of study participants were married (739, 98.8%) and protestants (594, 79.4%) in religion. Majority of the respondents were from Hadiya ethnic group (670, 89.6%) and 509 (68.0%) were rural residents.

Five hundred four (67.4%) of the respondents were in the age group of 25–34 years. In terms of educational status of women, 405 (54.1 %) had no formal education, 181 (24.2%) were primary level, and 162 (21.7%) attended secondary and above (Table 1).

**Table 1.** Sociodemographic characteristics of pregnant women interviewed in Hadiya zone south Ethiopia, 2017

**Variables**	**Frequency (n)**	**Percent (%)**
**Maternal age**		
15–24	182	24.3
25–34	504	67.4
35+	62	8.3
**Ethnicity**		
Hadiya	670	89.6
Kambata	34	4.5
Gurage	21	2.8
Amhara	11	1.5
Others^*^	12	1.6
**Marital status**		
Married	739	98.8
Single/Divorced/Widowed	9	1.2
**Residence**		
Rural	509	68.0
Urban	239	32.0
**Educational status**		
No formal education	405	54.1
Primary level (1–8)	181	24.2
Secondary and above (9–12) +	162	21.7
**Religion**		
Protestant	594	79.4
Orthodox	97	13.0
Muslim	42	5.6
Catholic	15	2.0
**Occupation**		
Housewife	626	83.7
Government employee	85	11.4
Others^**^	37	4.9
**Wealth index**		
Low	249	33.3
Middle	250	33.4
Upper	249	33.3

Note

* Silte, Oromo, Woleta, Halaba

** self-employee, student, daily laborers

### Access to health information or health services:

From a total of 748 respondents, 483 (64.6%) had exposure to mass media such as TV, radio and the rest (265, 35.4%) had no exposure to mass media. Concerning to distance to nearest health facility, 304 (40.6%) of respondents took thirty to sixty minutes, 295 (39.4%) of respondents took less than thirty minutes, and 149 (20.1%) of respondents took greater than one hour to walk on foot.

### Reproductive health related characteristics:

From the total pregnant women interviewed, 151 (20.2%) were primigravida (Gravida one), 437 (58.4%) were gravida one to four and 160 (21.4%) were gravida five and above. The median age of women in their first pregnancy was 21 years with IQR of 3. In this study, 474 (63.4%) women were participating in all household decision and the rest (36.6%) were not participating in all household decision.

### Prevalence of unintended pregnancy:

In this study, from the total pregnant women interviewed, 36.2% (95% CI; 32.9–39.63) experienced unintended pregnancy from which 230 (30.7%) experienced mistimed pregnancy and 41 (5.5%) had unwanted pregnancy.

### Unintended pregnancy and maternal antenatal care use during pregnancy:

Among the study participants, 73.1% (95% CI; 70.1–76.2) received at least one ANC visit during their pregnancy while the rest did not. Of those who received ANC, 343 (62.7%) received from health center and the rest (21.8% and 15.5%) were from hospital and health post, respectively. Only about 4.8% (n=26) had received the WHO recommended 4 or more ANC visits from skilled professionals. Besides the fourth antenatal care visit, 144 (26.3%), 249 (45.5%) and 128 (23.4%) pregnant women had first, second and third visits during the recent pregnancy, respectively. 84.6% of women with intended pregnancy and 52.8% of women with unintended pregnancy used ANC. Out of antenatal care attendants, 503 (92.0%) and 464 (84.6%) took the minimum recommended tetanus toxoid (TT) dose and iron folate supplementation, respectively.

When the use of antenatal care with different maternal and sociodemographic characteristics is concerned, the higher proportion of pregnant women with age group 15–24 years (138, 75%), women with secondary and above education (138, 85%), women who intended pregnancy (84.6%), women participating in all household decisions (81%), women with gravida one (85%), and women living nearer to health facility (86.4%) used antenatal care at least once as compared to their counterparts.

### Multivariate associations of unintended pregnancy and antenatal care use:

Factors that were associated with antenatal care use on bivariate analysis using enter method at the level of P value less than 0.25 were fit in multivariate logistic regression model. Accordingly, variables such as age, educational status, occupation, place of residence, wealth index, pregnancy intention, exposure to health information, participation in household decision, distance from health facility and gravidity were entered in to the multivariate logistic regression model using backward LR method.

The result showed that women with unintended pregnancy were 69% less likely to receive antenatal care from a health professional (AOR=0.31, 95% CI; 0.21–0.46) as compared to women with intended pregnancy after controlling other possible variables in the model.

Variables other than pregnancy intention were significantly associated with antenatal care use and they were maternal occupation, wealth index, participation in household decision and gravidity. Odds of ANC use were two times higher for government employers when compared with housewives (AOR=2.33, 95% CI; 1.01–5.35). Women from the middle wealth tertile were 43% less likely to receive antenatal care as compared to women from the lowest wealth tertile (AOR=0.57, 95% CI; 0.36–0.89). Women who participated in household decision making were 62% more likely to receive ANC than their counterparts (AOR= 1.62, 95% CI; 1.11–2.38). Women belonging to gravida five and more were 60% less likely to receive ANC services when compared with those belonging to gravida one (AOR=0.40, 95% CI; 0.22–0.74) (Table 2).

**Table 2.** Multivariate association of unintended pregnancy and antenatal care among pregnant women in Hadiya zone, southern Ethiopia, 2017

**Variable**	**ANC used (%)**	**ANC not used (%)**	**OR** **95% C.I for EXP(B)**	**AOR** **95% C.I for EXP(B)**
**Age**				
15–24	138(75.8)	44 (24.2)	1	1
25–34	372(73.8)	132 (26.2)	0.89 (0.60–1.33)	1.38 (0.84–2.28)
35+	37 (59.7)	25 (40.3)	0.47 (0.25–0.86)	1.21 (0.55–2.65)
**Educational status**				
No education	283(69.9)	122(30.1)	1	1
Primary (1–8)	126(69.6)	55 (30.4)	0.98 (0.67–1.44)	1.01(0.66–1.55)
Secondary and above (9–12)+	138(85.2)	24 (14.8)	2.47 (1.53–4.01)	0.99 (0.51–1.90)
**Occupation**				
Housewife	444(70.9)	182(29.1)	1	1
Government employee	78 (91.7)	7(8.3)	4.56 (2.06–10.08)	2.33 (1.01–5.35) *
Other	25 (67.6)	12(32.4)	0.85 (0.42–1.73)	0.90 (0.42–1.94)
**Wealth index**				
Lower	200(80.3)	49(19.7)	1	1
Middle	173(69.2)	77(30.8)	0.55 (0.36–0.83)	0.57 (0.36–0.89) *
Upper	174(69.9)	75(30.1)	0.56 (0.37–0.85)	0.63(0.40–1.0)
**Pregnancy intention**				
Intended	404(84.7)	73(15.3)	1	1
Unintended	143(52.8)	128(47.2)	0.20 (0.14–0.28)	0.31 (0.21–0.46)**
**Participated in decision**				
No	163(54.5)	111(44.5)	1	1
Yes	384(81)	90(19)	2.90 (2.08–4.05)	1.62 (1.11–2.38)*
**Exposure to mass media**				
Have no exposure	377(64.2)	106(35.8)	1	1
Have exposure	170(78.1)	95(21.9)	1.98 (1.42–2.76)	1.18(0.79–1.76)
**Distance from health facility**				
Less than 30 *min*	226(76.6)	69(23.4)	1	1
30–60 *min*	224(73.4)	80(26.6)	0.85 (0.59–1.23)	1.21(0.79–1.84)
Greater than one hour	97(65.1)	52(34.9)	0.57 (0.37–0.87)	0.98(0.58–1.66)
**Gravidity**				
Gravida 1	129(85.4)	22(14.6)	1	1
Gravida 2–4	325(74.4)	112(25.6)	0.49 (0.30–0.81)	0.74 (0.43–1.26)
Gravida 5+	93(58.1)	67(41.9)	0.23 (0.13–0.41)	0.40 (0.22–0.74)**
**Family size**				
1–4	261(80.5)	63(19.5)	1	1
5–8	264(67)	128(33)	0.49 (0.35–0.70)	0.86(0.55–1.35)
9–12	22(68.7)	10(31.3)	0.53 (0.23–1.17)	1.17(0.46–2.99)

* P<0.05

** P<0.01

### Time of ANC initiation and unintended pregnancy:

Even among the users of ANC, 85.4% (95% CI; 82.3–88.3) of women started their first antenatal visit lately after the first four months as a result (77.2% in the 2nd trimester and 8.2% in the 3rd trimester). The median gestational age at the first antenatal care visit was six months. Early ANC initiation was the highest for intended pregnancies but lowest for unintended pregnancies.

The result showed that pregnancy intention is significantly associated with delayed (late) ANC initiation. Women with unintended pregnancy were four times (AOR=4.40, 95% CI; 1.70–11.40) more likely to have delayed initiation of ANC when compared with intended pregnancy after controlling all the other variables.

Variables other than pregnancy intention were significantly associated with late initiation of antenatal care; exposure to mass media, distance from health facility, and gravidity or number of total pregnancy were the typical ones. Odds of late ANC initiation were two times (AOR=2.43, 95% CI; 1.17–5.02) more likely for women who had no exposure to mass media as compared to women who had exposure. Women who travel less than one hour to the nearest health facility were 52% less likely to late ANC initiation than those who travel more than one hour. Women with gravida five or more were 3.62 times (AOR= 3.62, 95% CI; 1.18–11.07) more likely to experience late ANC initiation when compared to gravida one women (Table 3).

**Table 3.** Multiple logistic regression model with backward to show association of unintended pregnancy and late initiation of ANC among pregnant women in Hadiya zone, southern Ethiopia, 2017

**Variable**	**ANC late initiation (%)**	**ANC early initiation (%)**	**COR** **95% C.I for EXP(B)**	**AOR** **95% C.I for EXP(B)**
**Age**				
15–24	107(77.5)	31(22.5)	1	1
25–34	326(87.6)	46(12.4)	2.05 (1.239–3.40)	1.64(0.86–3.10)
35+	34(91.9)	3(8.1)	3.28(0.94–11.41)	1.75(0.39–7.75)
**Educational status**				
No education	248(87.6)	35(12.4)	1	1
Primary (1–8)	114(90.5)	12(9.5)	1.34 (0.67–2.67)	1.21(0.58–2.52)
Secondary and above (9–12)+	105(76.1)	33(23.9)	0.44 (0.26–0.76)	0.72(0.40–1.28)
**Pregnancy intention**				
Intended	329(81.4)	75(18.6)	1	1
Unintended	138(96.5)	5(3.5)	6.29 (2.49–15.89)	4.40(1.70–11.40)^**^
**Exposure to mass media**				
Have no exposure	160(94.1)	10(5.9)	3.64 (1.83–7.27)	2.43 (1.17–5.02)^*^
Have exposure	307(81.4)	70(18.6)	1	1
**Distance from health facility**				
=< 1 *hr*	291(90.6)	30(9.4)	0.36 (0.22–0.59)	0.48 (0.28–0.80) ^**^
>1 *hr*	176(77.9)	50(22.1)	1	1
**Participated in decision**				
No	146(89.6)	17(10.4)	1	1
Yes	321(83.6)	63(16.4)	2.90 (2.08–4.05)	1.29(0.67–2.46)
**Gravidity**				
Gravida 1	100(77.5)	29(22.5)	1	1
Gravida 2–4	278(85.5)	47(14.5)	1.71 (1.02–2.87)	1.31 (0.76–2.26)
Gravida 5+	89(95.7)	4(4.3)	6.45 (2.18–19.07)	3.62 (1.18–11.07)^*^
**Family size**				
1–4	211(80.8)	50(19.2)	1	1
5–12	256(89.5)	30(10.5)	2.02 (1.24–3.29)	1.02(0.55–1.90)

* p<0.05,

** p<0.01

## Discussion

The study found significant association between unintended pregnancy and use of antenatal care among pregnant women in Hadiya zone, southern Ethiopia. The magnitude of unintended pregnancy in the study area was noticeably high in light of the goals of ensuring the women reproductive health and rights which was 36.2% among the study population, 30.7% for mistimed and 5.5% for unwanted pregnancy.

This finding was consistent with the study conducted in different parts of Ethiopia (13, 22–24) and reports of developing countries (2). The magnitude was higher than the study conducted in Gelemso General Hospital, Ethiopia (25). And the finding value was slightly lower than studies conducted in developed countries (2). This difference could be due to the fact that the latter study was conducted after child birth through interviews from most recent pregnancy but this study considers the time during pregnancy. And also the differences can be attributed to sociodemographic characteristics, and availability of health service.

In this study, 73.1% of women received at least one antenatal care visit during this pregnancy. This value was higher than the findings of the 2016 EDHS (5) and East Wollega Zone, Ethiopia (26), and also higher than multilevel analysis of Zambia (27). However, it was lower than the value in the study conducted in Hosanna town, Ethiopia (28). The difference could be due to EDHS including the remote areas while this study area might have increased access to the service. And also sociodemographic difference might be related to Zambia and the study in Hosanna town considered only urban individuals.

Additionally, about one in twenty (5%) women had received the WHO recommendation of 4 or more ANC visits from skilled professionals which was lower than 2016 EDHS (5) and East Wollega Zone (26). Even among users of ANC, 85.4% of women started the first ANC visit late after the first four months. The median of gestational age at the first antenatal care visit was six months. This finding was consistent with the studies done in Arba Minch Town and Arba Minch District, Ethiopia (29) and Ambo town, Ethiopia (30).

This study revealed that unintended pregnancy was significantly associated with ANC. Thus, the odds of receiving antenatal care were lower for women with unintended pregnancy as compared to women with intended pregnancy. And also, late initiation of ANC was higher for mothers with unintended pregnancy than mothers with intended pregnancy. Similarly, different studies conducted in developing and developed countries showed that women with unintended pregnancy do not use ANC or receive inadequate care (13, 15, 31–34). This could be due to those women who were less prepared financially and emotionally for the demands of pregnancy and childbearing (35) and also, most likely due to the delay in recognizing pregnancy.

Furthermore, in this study, other factors independently associated with ANC use included maternal occupation, wealth, and gravidity. Government employers were more likely to receive ANC when compared with housewives. This finding was supported by other studies done in North West Ethiopia (36) and Kenya (32). The possible justification could be that women who are employed might have a better access and understanding of the ANC services. Women who participated in household decision making were more likely to receive ANC than women who did not participate which was similar with the study done in southwestern Ethiopia (12). This might be due to the fact that when resources are controlled by women, they might have the freedom to use the service whenever they need it. Women who belong to gravida five or more were less likely to receive ANC than gravida one. This was in line with study in rural Nepal that mothers in the first pregnancy had higher ANC visit than second or higher number of pregnancies (37). The reason could be due to being confident about pregnancy from previous experience which may reduce maternal health seeking behavior.

Women’s exposure to mass media, gravidity, and distance from health facility were significantly associated with late ANC initiation. Women who had no exposure to mass media were more likely to have late ANC initiation than their counterparts which was in agreement with the study in Ambo town, Ethiopia (30) and Nigeria (38). Late initiation of ANC was 3 times more in gravida 5+ women as compared to gravida one women. This was supported by the study conducted in Wollaita Soddo town, Ethiopia and it indicated that, pregnant women who have no history of parity were more likely to initiate ANC visit timely than women with one and above (39).

### Limitation of this study:

This study has its own limitations. There is social desirability bias since it is self-report. Temporal relationships of the outcome variable and the predictor variables cannot be established due to cross sectional nature of the study.

## Conclusion

This study found significant association in unintended pregnancy and maternal behavior during pregnancy in the study area. More than one-third of women had unintended pregnancy in the area. The magnitude of unintended pregnancy was nearly comparable with developed countries. And also, women with unintended pregnancy were less likely to use ANC, and more likely to have delayed initiation of ANC.

Ministry of Health, Regional Health Bureau, Zonal Health Department and Woreda Health Office should strengthen the prevention of unintended pregnancy through utilization of effective contraception that will help to reduce the magnitude of these unhealthy prenatal behaviors. Also, majority of the mothers who attend ANC initiated the visits later than the time recommended by World Health Organization.

Therefore, behavioral change communication (BCC) is recommended at individual and community level in attaining healthy behaviors during pregnancy. Health professionals, particularly Health Extension Workers (HEWs), are placed to counsel especially those women who happen to conceive unintentionally in order to minimize the risk of having unhealthy behavior like not using ANC or receiving inadequate care and delaying ANC initiation.

Longitudinal study including husband’s information will be suggested on pregnancy intention and maternal behavior during pregnancy in the study area.
